# Synthesis of Flower-Like AgI/BiOCOOH p-n Heterojunctions With Enhanced Visible-Light Photocatalytic Performance for the Removal of Toxic Pollutants

**DOI:** 10.3389/fchem.2018.00518

**Published:** 2018-10-26

**Authors:** Shijie Li, Wei Jiang, Kaibing Xu, Shiwei Hu, Yu Liu, Yingtang Zhou, Jianshe Liu

**Affiliations:** ^1^Key Laboratory of Key Technical Factors in Zhejiang Seafood Health Hazards, Institute of Innovation and Application, Zhejiang Ocean University, Zhoushan, China; ^2^State Key Laboratory for Modification of Chemical Fibers and Polymer Materials, Research Center for Analysis and Measurement, Donghua University, Shanghai, China; ^3^State Environmental Protection Engineering Center for Pollution Treatment and Control in Textile Industry, College of Environmental Science and Engineering, Donghua University, Shanghai, China

**Keywords:** AgI/BiOCOOH, p-n heterojunction, photocatalysis, dye removal, antibiotic removal

## Abstract

In this study, flower-like AgI/BiOCOOH heterojunctions were constructed through a two-step procedure involving the solvothermal synthesis of BiOCOOH microflowers followed by AgI modification using a precipitation method. These novel photocatalysts were systematically examined by XRD, UV–vis DRS, SEM, TEM, EDS, and PL spectroscopy techniques. The AgI/BiOCOOH heterojunction were studied as a decent photocatalyst for the removal of the industrial dye (rhodamine B, and methyl blue) and antibiotic (tetracycline) under visible light. The AgI/BiOCOOH heterojunctions are much more active than bare BiOCOOH, and AgI, which could be ascribed to the improved separation of charge carriers, resulting from the formation of p-n heterojunction between two constituents. The holes (h^+^) and superoxide radical (•O${}_{2}^{-}$) were detected as the main active species responsible for the pollutant degradation. The results showed that a highly efficient visible-light-driven photocatalytic system was developed for the decomposition of toxic pollutants.

**HIGHLIGHTS**

- Novel architectures of AgI nanoparticles anchored on BiOCOOH micro-flowers were prepared.
- Novel AgI/BiOCOOH p-n heterojunction displayed excellent photocatalytic performance.
- AgI/BiOCOOH heterojunctions significantly enhance the visible-light absorption and boost the charge separation.

## Introduction

Harvesting sunlight to degrade industrial pollutants in wastewater is an effective and sustainable technology for environmental purification (Chong et al., 2010; Zhu and Wang, 2017). The key of the photocatalysis technique is to develop catalysts with superior photocatalytic performance (Zhang et al., 2015; Ji et al., 2017; Wang and Astruc, 2017; Zhu and Wang, 2017; Moroz et al., 2018; Wu et al., 2018). Till now, various photocatalysts have been constructed and studied (Zhang et al., 2012, 2016; Wang and Astruc, 2017; Mousavi et al., 2018). Among them, Bi-based semiconductors have been regarded as excellent photocatalysts by virtue of its decent photocatalytic activity, strong photo-redox driving force, high chemical stability and nontoxicity (Ahern et al., 2015). However, superior photo-redox force is commonly accompanied with a wide band gap (*E*_g_), which substantially restrains the visible-light absorption. Therefore, the enhancement of visible-light absorption of Bi-based photocatalysts is quite of necessity (Li et al., 2018a,b; Lin et al., 2018).

Various strategies have been applied to ameliorate the visible-light response of Bi-based semiconductors through metal/nonmetal doping (Wu et al., 2017; Li et al., 2018d,e), architecture tailoring (Xiong et al., 2011; Li et al., 2016), heterojunction constructing (Li et al., 2018c; Zhang and Ma, 2018). In particular, the combination of Bi-based semiconductors with narrow band gap semiconductors is an effective way to improve the photocatalytic performance, due to the improved visible-light absorption and boosted separation of electron-hole pairs at the interface (Li et al., 2017d).

BiOCOOH, as a type of layered Bi-based oxides, is composed of [Bi_2_O_2_]^2+^ fluorite-like layers intercalating by formic acid (Wei et al., 2015; Xu et al., 2016, 2018; Chen et al., 2018). However, the wide band gap (*E*_g_ = 3.4 eV) of BiOCOOH makes it only active under ultraviolet light irradiation, which severely limits its photocatalytic performance (Xu et al., 2018). The construction of BiOCOOH-based heterojunctions is advantageous for improving the visible-light photocatalytic performance of BiOCOOH (Chai and Wang, 2015; Chen et al., 2016). As a result, some BiOCOOH-based heterojunctions have been constructed and exhibit better photocatalytic activity under visible light (Chai and Wang, 2015; Li et al., 2017e,f; Chen et al., 2018). The further exploration of novel BiOCOOH-based heterojunction catalysts **i**s still required to provide more potential candidates for practical application and to reveal the reasons for the synergy effect between the constituents.

Recently, AgI has proved a good photosensitizer due to the fact that its narrow band gap (~2.7 V) is in favor of the light absorption of semiconductors (Wang et al., 2016; Liang et al., 2017, 2018; Xiang et al., 2018). Hence, it inspires us to combine BiOCOOH with AgI for achieving novel AgI/BiOCOOH p-n heterojunctions with distinguished visible-light photocatalytic performance.

In this study, we have successfully fabricated flower-like AgI/BiOCOOH heterojucntions for the first time, through a simple precipitation approach at room temperature. Substantial improvement of the visible-light harvesting ability can be observed after the introduction of AgI. Meanwhile, the novel p-n heterojunction between AgI and BiOCOOH ensures the effective separation of photo-generated electron and holes. As a result, the AgI/BiOCOOH heterojucntions exhibited much higher activity in the photocatalytic degradation of rhodamine B (RhB), and methyl blue (MB), and tetracycline hydrochloride (TC) antibiotic under visible light. This work offers a simple way to improve the photocatalytic activity of BiOCOOH toward pollutant removal by coupling with AgI.

## Experiment

### Chemicals

Bi(NO_3_)_3_•5H_2_O, glycerol, N,N-dimethyformamide (DMF), methyl blue (MB), tetracycline hydrochloride (TC), rhodamine B (RhB), AgNO_3_, KI, iso-propanol (IPA), ammonium oxalate (AO), and *p*-benzoquinone (BQ) were bought from Chemical Reagent factory (China).

### Synthesis

BiOCOOH microspheres were prepared via a solvothermal method. Briefly, Bi(NO_3_)_3_•5H_2_O (4 mmol) was dissolved in the solution of 50 mL of glycerol, 20 mL of DMF and 10 mL of H_2_O with the assistance of ultra-sonication. After being stirring for 1 h, the solution was poured into a 100 mL autoclave and kept at 160°C for 20 h. When the reaction finished and the system was cooled down, the white precipitants separated from the suspension were washed with deionized water and ethanol for several times, and dried at 70°C overnight.

Flower-like AgI/BiOCOOH heterojunctions were fabricated by a simple *in situ* precipitation method at room temperature. Firstly, 1 mmol BiOCOOH was dispersed in 50 mL of deionized water containing 0.1 mmol AgNO_3_ to form a homogeneous suspension and kept stirring for 2 h in darkness. Secondly, 10 mL of KI solution (0.01 M) was added into the above suspension slowly under continuously stirring in darkness. After being stirring for 3 h, the obtained solid named as S1 was washed, and dried at 70°C overnight. By varying the addition amount of AgNO_3_ and KI, the other heterojunctions with various AgI/BiOCOOH molar ratios (0.5/1, 1/1, and 1.5/1) were fabricated and named as S2, S3, and S4, respectively.

### Characterization

X-ray diffraction (XRD) patterns of the samples were collected on a Bruker D8 Advance diffractometer with Cu Kα radiation. The scanned range of 2θ was from 20° to 80°. Hitachi S−4800 scanning electron microscope (SEM), and energy dispersive X-ray spectroscopy (EDX), and JEM−2010F transmission electron microscope were used to investigate the microstructures and compositions of the samples. The UV-vis diffused reflectance spectra (DRS) were recorded on a Shimadzu UV−2600 UV–vis spectrophotometer. The photoluminescence (PL) spectra were recorded on a Hitachi F-7000 fluorescence spectrophotometer. The Brunauer-Emmett-Teller (BET) surface areas were determined by a Micromeritics ASAP 2020 equipment.

### Photocatalytic tests

The photocatalytic degradation of RhB, MB, or TC was executed to evaluate the activity of catalysts. Typically, 30 mg of catalyst was first added into 100 mL of RhB (10 mg L^−1^), MB (10 mg L^−1^), or TC (20 mg L^−1^) solution in a reactor and then stirred for half an hour in the dark. Subsequently, the photocatalytic reaction was initiated when a 300 W xenon lamp coupled with a UV-cutoff filter (λ > 400 nm) switched on, 2 mL of the suspensions were extracted at certain intervals, and separated by centrifugation to get the supernatant solutions. The concentrations of solutions were determined by using a UV−2600 spectrophotometer. Total organic carbon (TOC) experiments were conducted by the photocatalytic degradation of RhB (40 mg L^−1^, 150 mL) solution over 200 mg of S3.

## Results and discussion

### Characterization

**Figure 1:**
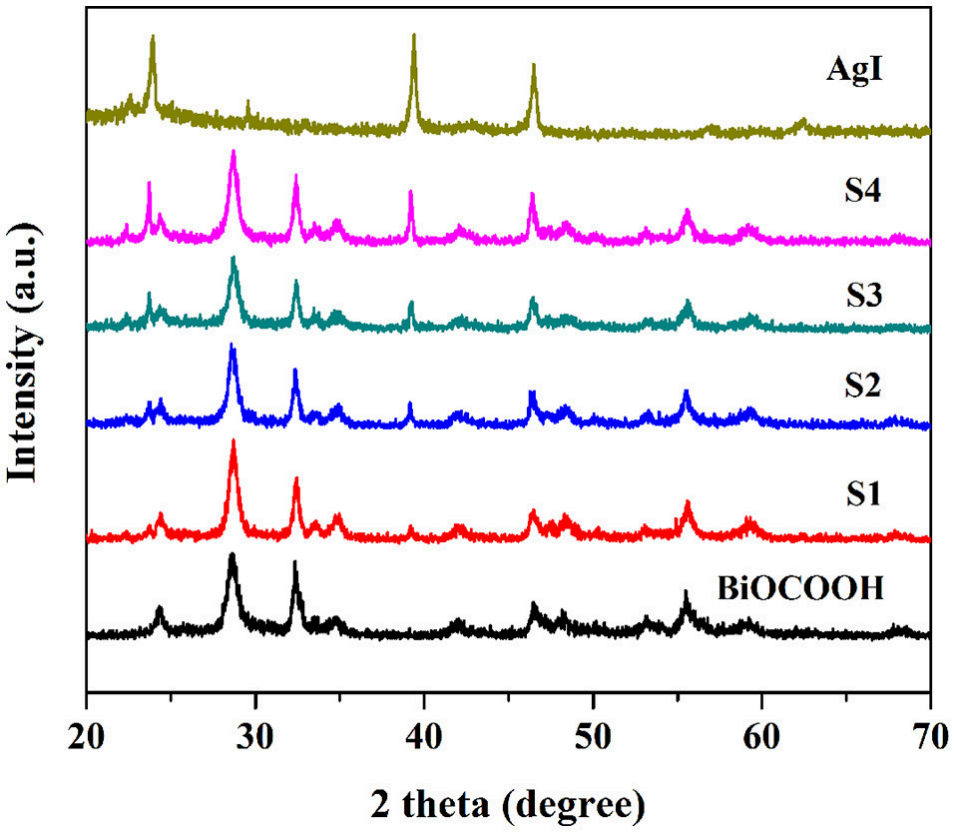
XRD patterns of AgI/BiOCOOH heterojunctions (S1, S2, S3, and S4), pristine BiOCOOH and AgI.

A series of AgI/BiOCOOH heterojunctions with different molar ratios (0.1/1, 0.5/1, 1/1, and 1.5/1) were prepared and denoted as S1, S2, S3, and S4, respectively. AgI, BiOCOOH, and AgI/BiOCOOH heterojunctions (S1, S2, S3, and S4) were characterized by XRD and the XRD patterns are shown in Figure 1. Pure AgI and BiOCOOH could be indexed as the miersite AgI (JCPDS 78-0641) and the tetragonal BiOCOOH (JCPDS 35-0939), respectively. The XRD patterns of AgI/BiOCOOH heterojunctions generally resemble that of BiOCOOH, but some peaks belonging to AgI are also detected, indicating the co-existence of AgI and BiOCOOH in these heterojunctions.

**Figure 2:**
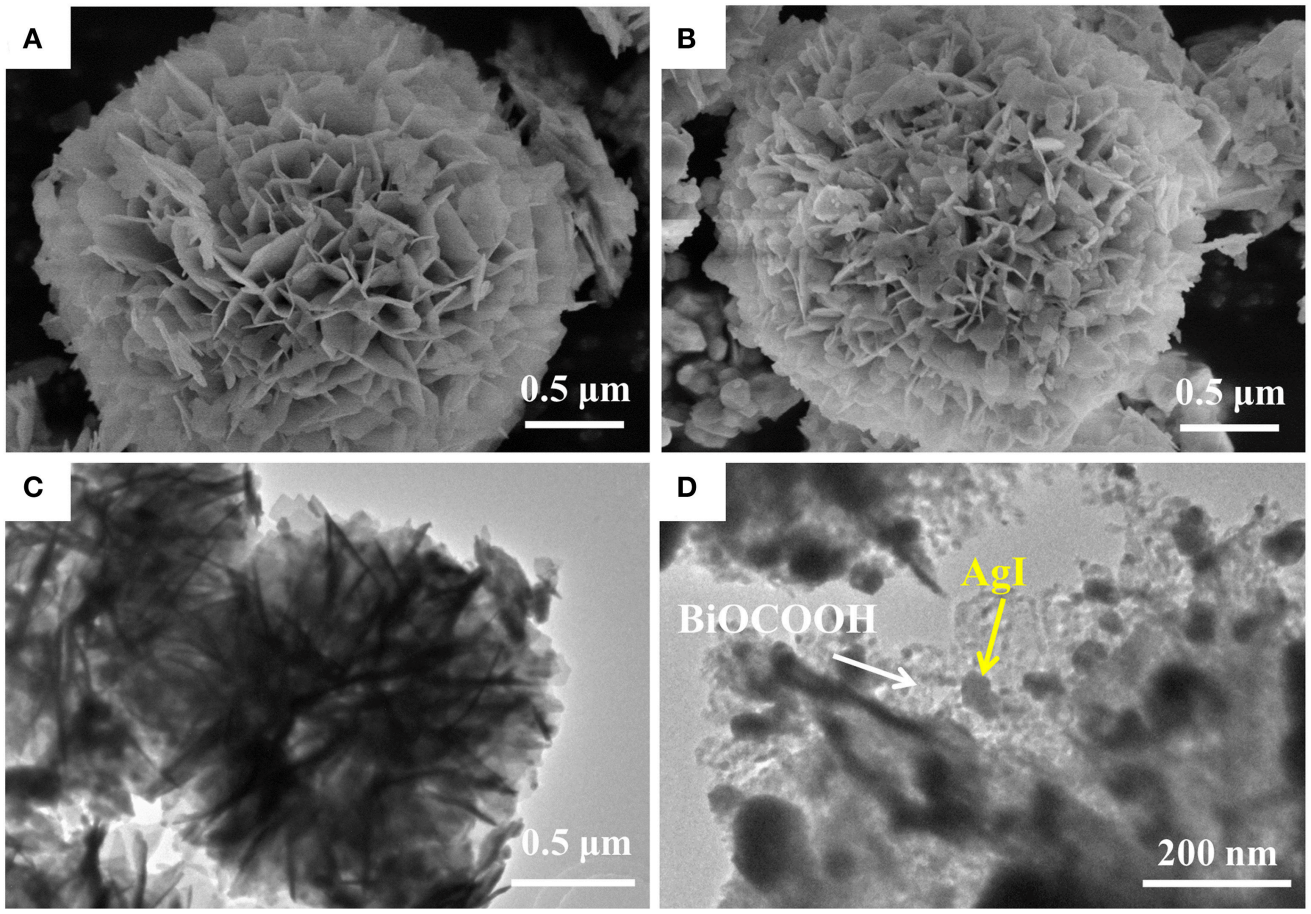
SEM images of bare BiOCOOH **(A)** and S3 **(B)**; TEM images of S3 **(C,D)**.

**Figure 3:**
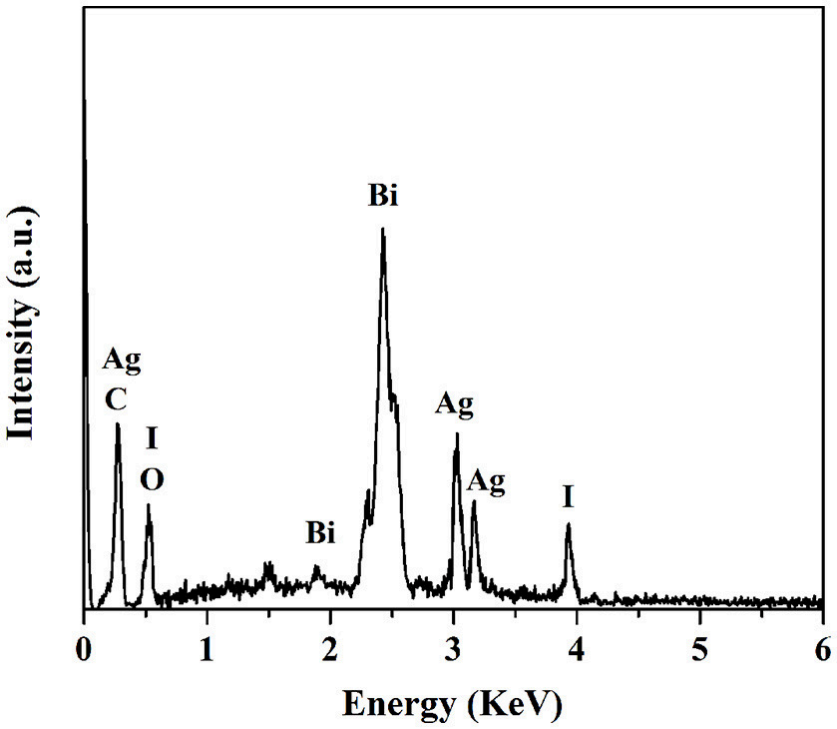
EDS spectra of S3.

The microstructure and morphology of BiOCOOH and the AgI/BiOCOOH heterojunction were surveyed by SEM and TEM (Figure 2). Obviously, bare BiOCOOH exhibits hierarchical structures assembled from numerous nano-sheets with size of 150–500 nm (Figure 2A). After the introduction of AgI, the as-prepared AgI/BiOCOOH maintained the microsphere structure. Representatively, S3 consists of flower-like BiOCOOH spheres and AgI nanoparticles (size: 20–150 nm, Figure 2B), in which AgI nanoparticles are deposited on BiOCOOH spheres. The detailed structural information was further obtained by the TEM images. Figures 2C,D confirms that AgI nanoparticles and BiOCOOH spheres are attached to each other, indicating the successful preparation of AgI/BiOCOOH heterojunction with a well contacted interface. Additionally, the energy dispersive spectroscopy (EDS) spectra reveals the presence of chemical elements Ag, I, C, O, and Bi in S3 (Figure 3).

**Figure 4:**
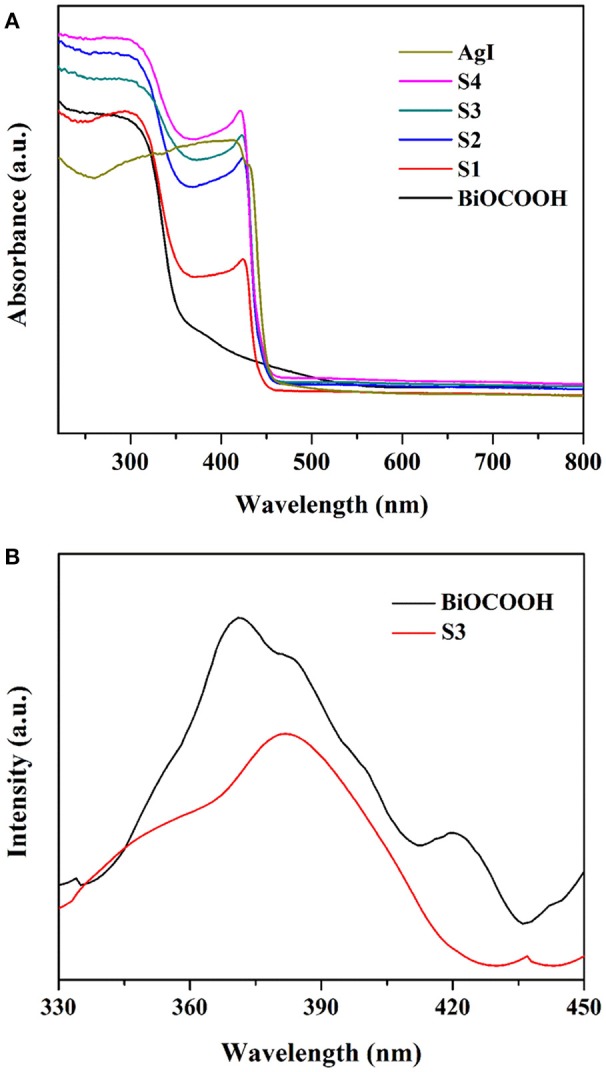
**(A)** UV–Vis DRS of bare BiOCOOH, AgI, and AgI/BiOCOOH heterojunctions (S1, S2, S3, and S4). **(B)** PL spectra of pristine BiOCOOH and S3 with an excitation wavelength of 300 nm.

For photocatalysts, the optical absorption behavior is recognized as one of the most significant factors in determining their photocatalytic activity (Akhundi and Habibi-Yangjeh, 2017; Li et al., 2017c; Wang et al., 2018). Thus, the optical absorption properties of BiOCOOH, AgI and the AgI/BiOCOOH heterojunctions were surveyed by UV–Vis diffuse reflection spectra (UV–Vis DRS) and the results are presented in Figure 4A. The absorption edges of pure AgI and BiOCOOH are about 450 nm (*E*_g_ = 2.76 eV) (Wang et al., 2016; Liang et al., 2017) and 365 nm (*E*_g_ = 3.40 eV) (Chai and Wang, 2015; Li et al., 2017f), respectively, in accordance with the reported results (Chai and Wang, 2015; Li et al., 2017f). Compared to pure BiOCOOH, AgI/BiOCOOH heterojunctions are endowed with intensive visible-light absorption.

The band potential of AgI and BiOCOOH could be calculated by the following equations:

(1) $$\begin{array}{r} {\text{E}}_{\text{VB}}=\text{X}-{\text{E}}_{0}+0.5{\text{E}}_{\text{g}} \end{array}$$

(2) $$\begin{array}{r} {\text{E}}_{\text{CB}}={\text{E}}_{\text{VB}}-{\text{E}}_{\text{g}} \end{array}$$

Where, the *X* represents the absolute electronegativity of the semiconductor. *E*_0_ value is ~4.5 eV. *E*_g_ means the band gap of the semiconductor. Hence, the *E*_CB_ and *E*_VB_ of AgI are calculated to be −0.40 and 2.36 eV, and those of BiOCOOH are −0.67 and 2.73 eV.

The separation efficiency of charge carriers is strongly related to the photocatalytic performance of the catalyst (Li et al., 2017a; Zhang et al., 2017). Thus, the PL technique was employed to probe the separation efficiency of electrons and holes. As shown in Figure 4B, BiOCOOH displays a main emission peak centered at ca. 370 nm. Intriguingly, S3 exhibits a much lower PL intensity than pristine BiOCOOH, illustrating that the formation of AgI/BiOCOOH heterojunction favors the separation of photo-induced electrons and holes.

### Photocatalytic property

**Figure 5:**
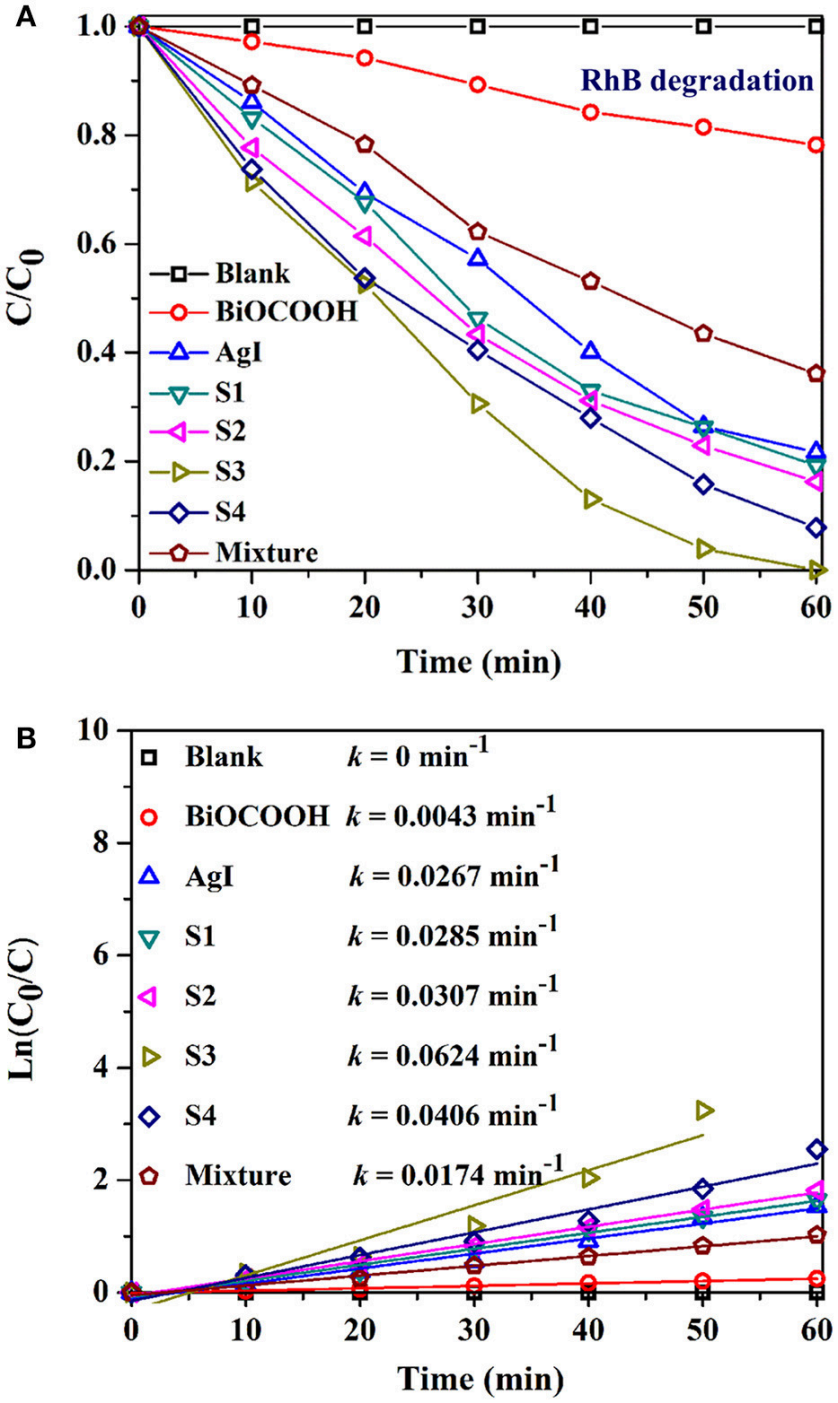
**(A)** Photocatalytic degradation of RhB over various catalysts under visible light. **(B)** The kinetic curves over different catalysts.

The photocatalytic activity of the AgI/BiOCOOH heterojunctions were tested through the degradation of industrial dyes (RhB, and MB), and antibiotic (TC) under visible light. Figure 5A shows the RhB degradation curves. No RhB degradation happened in the absence of catalysts. Only 21.8 or 78.3% of RhB degradation could be reached in 60 min when pristine BiOCOOH or AgI presented. Intriguingly, a synergistic effect is found between AgI and BiOCOOH when they are combined, endowing these heterojunctions with higher activity than that of pristine AgI or BiOCOOH. Among these AgI/BiOCOOH heterojunctions, S3 showed the best photocatalytic activity with 100% of RhB degraded within 60 min. Of note, the molar ratio of AgI to BiOCOOH plays a crucial role in the photocatalytic activity of the heterojunction. A much higher or lower molar ratio is not favorable for improving the activity of these heterojunctions. Besides, the BET surface areas of the catalysts were investigated and the results were listed in Table S1. It can be seen that the BET surface areas of BiOCOOH, S1, S2, S3, and S4 are 26.13, 28.27, 24.26, 20.72, and 15.21 m^2^g^−1^, respectively. Though S3 shows the highest activity, its BET surface area is not the largest among these samples. Obviously, the surface area does not play a dominant role in determining the photocatalytic performance of AgI/BiOCOOH.

To further explore the synergistic effect, the photocatalytic activity of the physical mixture (46.5 wt% AgI + 53.5 wt% BiOCOOH) was also measured and the RhB degradation efficiency in 60 min is much lower than that by using S3 as the catalyst, demonstrating that the establishment of p-n heterojunction favors the activity enhancement.

The kinetic behaviors of the RhB degradations in the presence of various catalysts also have been studied. The RhB degradation process can be fitted well by the pseudo-first–order model. As shown in Figure 5B, S3 achieves the highest k value of 0.0624 min^−1^, 13.5, 1.3, or 2.6 folds greater than that of bare BiOCOOH (0.0043 min^−1^), AgI (0.0267 min^−1^), or the mixture (0.0174 min^−1^).

**Figure 6:**
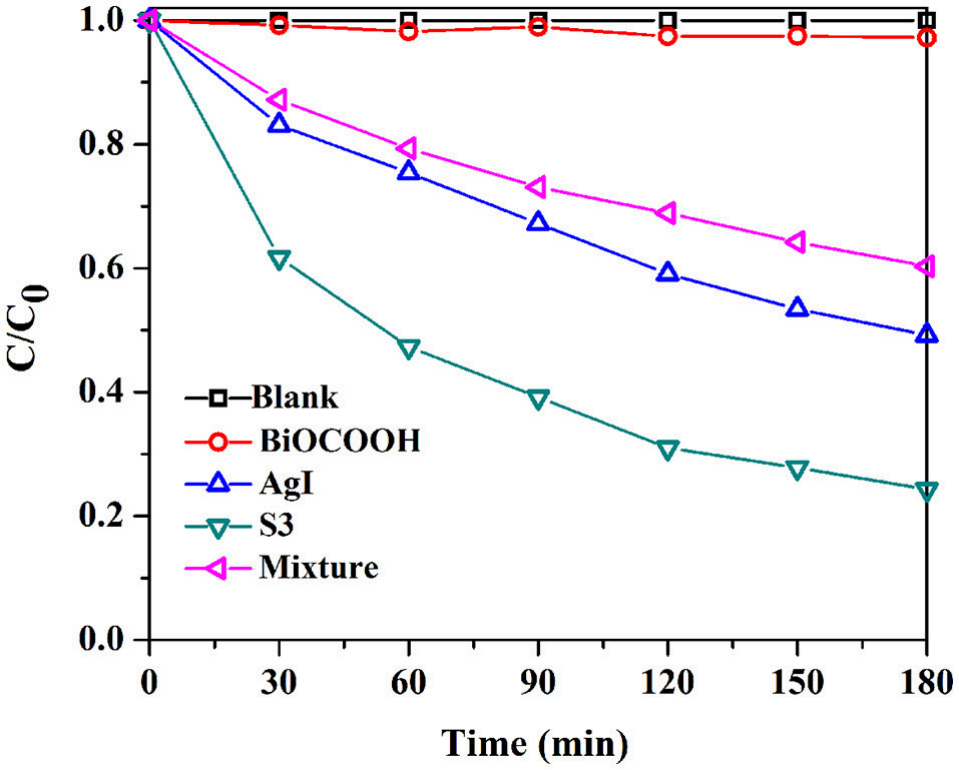
The TC degradation curves over various catalysts under visible light.

MB dye, and TC antibiotic, two types of toxic pollutants were also employed to further assess the photocatalytic performance of AgI/BiOCOOH (Figure 6, Figure S1). Attractively, the MB degradation efficiency achieved by using S3 reaches 85.2% in 90 min of reaction, much better than that achieved by employing pure AgI (68.7%), BiOCOOH (32.8%), or a physical mixture (60.2%) as the catalyst. In addition, a similar phenomenon is found when employing TC (Figure 6) as a target pollutant. The optimized S3 showed an excellent photocatalytic activity with TC degradation efficiency of 75.7% in 3 h, considerably higher than those of bare BiOCOOH (2.8%) and AgI (50.7%) as well as their mixtures (39.6%).

**Figure 7:**
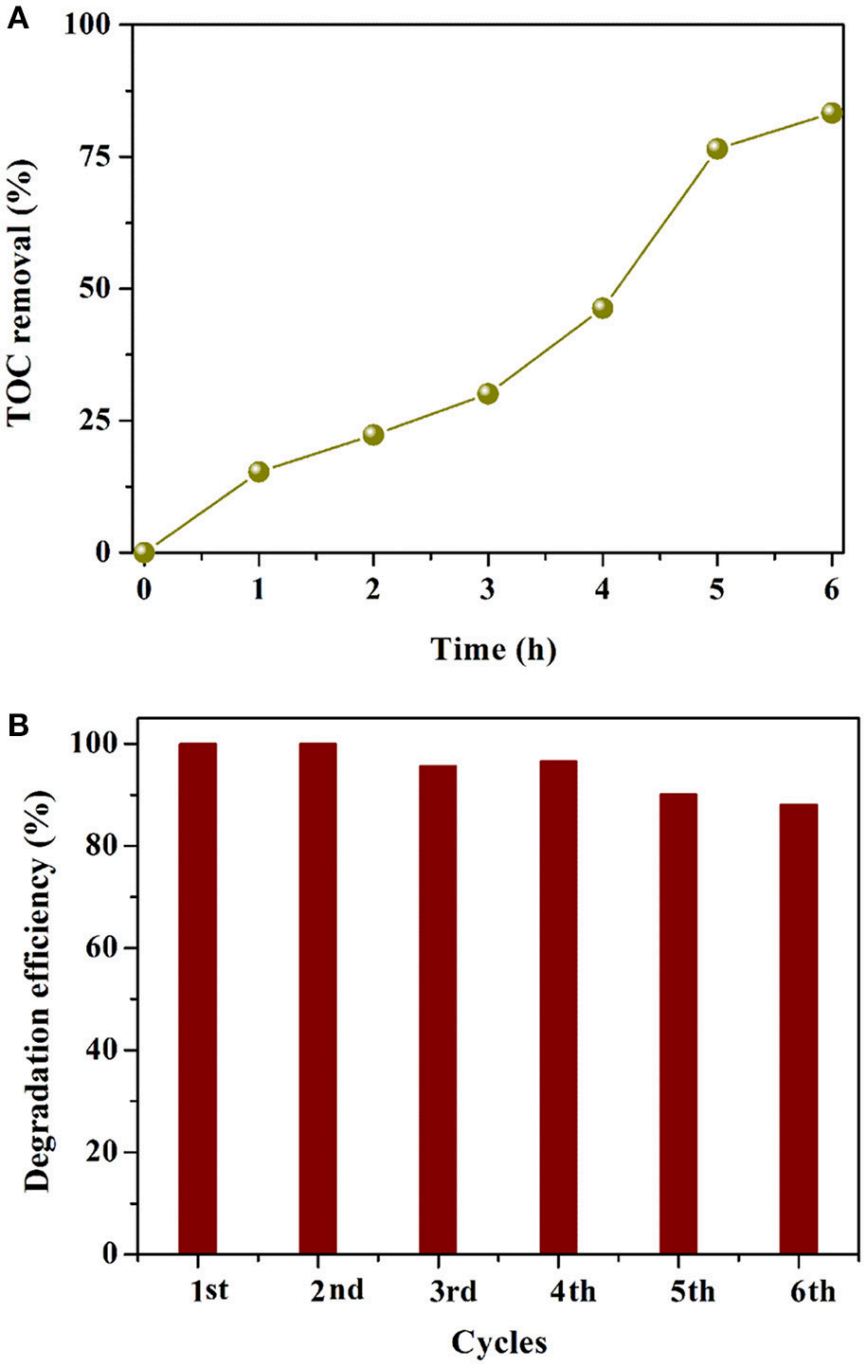
**(A)** TOC removal curves in the presence of S3. **(B)** Cycling runs in the degradation of RhB over S3.

To assess the mineralization ability of AgI/BiOCOOH, the TOC concentration during RhB (150 mL, 40 mg L^−1^) degradation by S3 (200 mg) was measured and analyzed. As illustrated in Figure 7A, notably, the TOC removal efficiency increases with the prolonging of reaction time and the final TOC mineralization efficiency yields 83.4% in 6 h, suggesting that AgI/BiOCOOH has the great potential for the deep treatment of toxic pollutants.

The stability of AgI/BiOCOOH (S3) was tested via recycling for six runs in the degradation of RhB. As depicted in Figure 7B, the RhB degradation efficiency at 60 min of the first run is 100%, while that value becomes 88.1% in the sixth run. The slight decline in the activity may be due to the catalyst loss in the recycling experiments. Besides, a small amount of Ag^0^ was produced after the cycling tests, which has been proved by the XRD results (Figure S2). It has been demonstrated that Ag/AgI displays a stable structure, which can facilitate the separation of charge carriers (Ren et al., 2018; Yuan et al., 2018). These results verify that AgI/BiOCOOH (S3) possesses good stability.

### The photocatalytic mechanism

**Figure 8:**
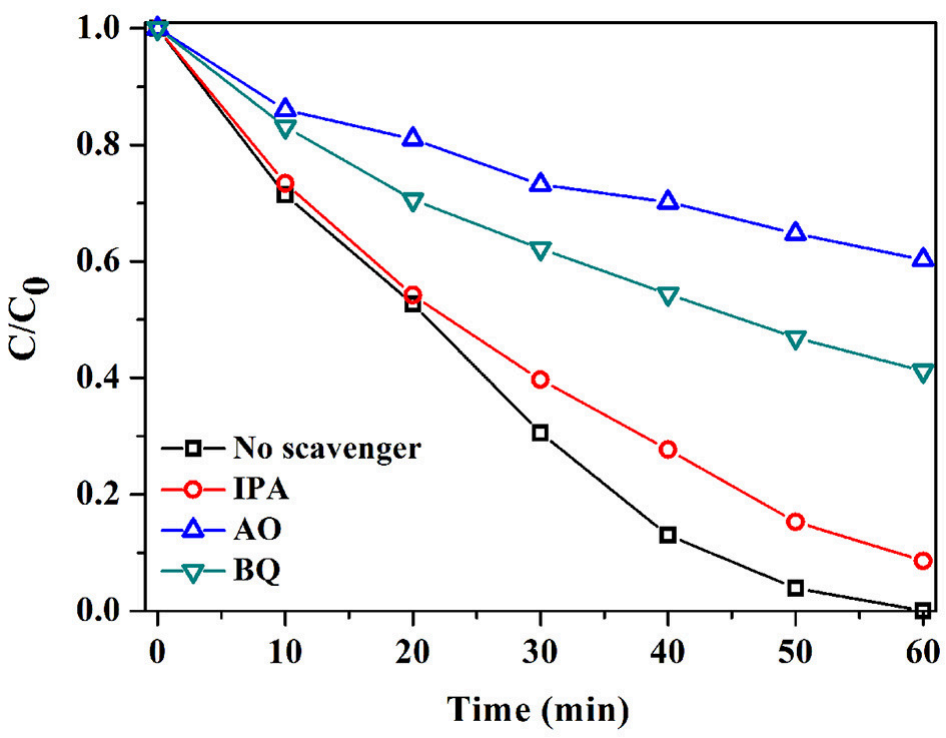
Radical-scavenge tests in the degradation of RhB over S3.

Radicals generated during the photocatalytic reaction are mainly involved in the degradation of pollutant (Wang et al., 2011). Therefore, to figure out their role in the degradation of RhB, radical-scavenge experiments were performed. As Figure 8 reveals, with the introduction of isopropanol (IPA), only a slight decrease in the activity of S3 was observed, with a RhB degradation efficiency of 99.14%. The result signified that •OH showed a negligible effect on RhB degradation. Instead, the addition of benzoquinone (BQ) and ammonium oxalate (AO) substantially inhibited the degradation of RhB, with RhB degradation efficiencies of only 58.8 and 39.7%. The results demonstrated the significant roles of •O${}_{2}^{-}$ and h^+^ in photocatalytic degradation of RhB over AgI/BiOCOOH.

**Figure 9:**
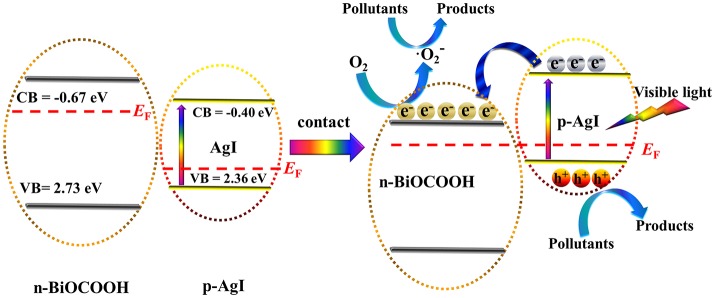
Proposed mechanism of pollutant degradation over AgI/BiOCOOH under visible light.

Based on the above analysis, a possible mechanism for the degradation of pollutants over AgI/BiOCOOH under visible light is brought forward as outlined in Figure 9. As shown in Figure 9, the Fermi level (*E*_F_) and conduction band (CB) of *p*-type AgI are lower than those of *n*-type BiOCOOH before they are in contact. After the contact between *p*-type AgI and *n*-type BiOCOOH, the *E*_F_ of AgI shifts upward, while that of BiOCOOH shifts downward until the *E*_F_ of both semiconductors achieves an equilibrium. Correspondingly, the CB and VB of AgI moves up along with the *E*_F_, and those of BiOCOOH moves down. As a consequence, the CB and VB of BiOCOOH are lower than those of AgI. Similar phenomenon was also observed in other p-n heterojunction photocatalysts (Li et al., 2017b). Under visible-light irradiation, AgI is excited to produce photo-generated electrons and holes on the CB and VB. The photo-generated electrons on the CB of AgI can easily drift to the CB of BiOCOOH, which is beneficial to the separation of electrons and holes (Ye et al., 2018), as proved by the PL results (Figure 4B). Subsequently, the electrons enriched on the CB of BiOCOOH can be captured by O_2_ to yield •O${}_{2}^{-}$ radical. Finally, the produced •O${}_{2}^{-}$ and h^+^ reactive species are involved in the degradation of toxic pollutants (RhB/MB/TC). Apparently, the construction of p-n heterojunction between *p*-type AgI and *n*-type BiOCOOH is favorable for the separation of electron-hole pairs, further enhancing the photocatalytic performance.

## Conclusions

AgI nanoparticles interspersed-BiOCOOH hetero-structures were prepared by a simple method. The visible-light harvesting ability is substantially improved with the introduction of AgI, compared with pristine BiOCOOH. In the photocatalytic pollutant (RhB, MB, and TC) degradation, the AgI/BiOCOOH heterojunctions, especially S3, displayed much higher photocatalytic activity than pristine BiOCOOH and AgI. Moreover, S3 possesses good reusability. The synergy effect on photocatalytic performance can be ascribed to the p-n heterostructure with improving visible-light absorption and promoting the separation of electron and holes. This work indicates that the well-designed AgI/BiOCOOH heterojunction could be potentially employed to remedy environment.

## Author contributions

SL designed and carried out the experiments, and analyze the data. SH, WJ, YL, KX, YZ, and JL assisted with part of the experiments. SL finished the manuscript. All authors have read and agreed the submission of the manuscript.

### Conflict of interest statement

The authors declare that the research was conducted in the absence of any commercial or financial relationships that could be construed as a potential conflict of interest.

## References

[B1] Ahern J. C. Fairchild R. Thomas J. S. Carr J. Patterson H. H. (2015). Characterization of BiOX compounds as photocatalysts for the degradation of pharmaceuticals in water. Appl. Catal. B-Environ. 179, 229–238. 10.1016/j.apcatb.2015.04.025

[B2] Akhundi A. Habibi-Yangjeh A. (2017). Graphitic carbon nitride nanosheets decorated with CuCr_2_O_4_ nanoparticles: novel photocatalysts with high performances in visible light degradation of water pollutants. J. Colloid Interface Sci. 504, 697–710. 10.1016/j.jcis.2017.06.025 28622563

[B3] Chai B. Wang X. (2015). Enhanced visible light photocatalytic activity of BiOI/BiOCOOH composites synthesized via ion exchange strategy. RSC Adv. 5, 7589–7596. 10.1039/C4RA10999F

[B4] Chen L. He J. Liu Y. Chen P. Au C.-T. Yin S.-F. (2016). Recent advances in bismuth-containing photocatalysts with heterojunctions. Chin. J. Catal. 37, 780–791. 10.1016/S1872-2067(15)61061-0

[B5] Chen P. Zhang Q. X. Su Y. H. Shen L. Z. Wang F. L. Liu H. J. (2018). Accelerated photocatalytic degradation of diclofenac by a novel CQDs/BiOCOOH hybrid material under visible-light irradiation: dechloridation, detoxicity, and a new superoxide radical model study. Chem. Eng. J. 332, 737–748. 10.1016/j.cej.2017.09.118

[B6] Chong M. N. Jin B. Chow C. W. K. Saint C. (2010). Recent developments in photocatalytic water treatment technology: a review. Water Res. 44, 2997–3027. 10.1016/j.watres.2010.02.039 20378145

[B7] Ji T. Cui Z. Zhang W. Cao Y. Zhang Y. He S.-A.. (2017). UV and visible light synergetic photodegradation using rutile TiO_2_ nanorod arrays based on a p-n junction. Dalton Trans. 46, 4296–4302. 10.1039/C7DT00261K 28281717

[B8] Li S. Hu S. Jiang W. Liu Y. Liu Y. Zhou Y.. (2018a). Ag_3_VO_4_ nanoparticles decorated Bi_2_O_2_CO_3_ micro-flowers: an efficient visible-light-driven photocatalyst for the removal of toxic contaminants. Front. Chem. 6:255. 10.3389/fchem.2018.00255 30013966 PMC6036280

[B9] Li S. Hu S. Jiang W. Zhou Y. Liu J. Wang Z. (2018b). Facile synthesis of cerium oxide nanoparticles decorated flower-like bismuth molybdate for enhanced photocatalytic activity toward organic pollutant degradation. J. Colloid Interface Sci. 530, 171–178. 10.1016/j.jcis.2018.06.084 29982008

[B10] Li S. Jiang W. Hu S. Liu Y. Liu Y. Xu K. (2018d). Hierarchical heterostructure of Bi_2_MoO_6_ micro-flowers decorated with Ag_2_CO_3_ nanoparticles for efficient visible-light-driven photocatalytic removal of toxic pollutants. Beilstein J. Nanotechnol. 9, 2297–2305. 10.3762/bjnano.9.214 30202698 PMC6122119

[B11] Li S. Shen X. Liu J. Zhang L. (2017a). Synthesis of Ta_3_N_5_/Bi_2_MoO_6_ core-shell fiber-shaped heterojunctions as efficient and easily recyclable photocatalysts. Environ. Sci. Nano 4, 1155–1167. 10.1039/C6EN00706F

[B12] Li S. J. Hu S. W. Jiang W. Liu Y. Liu J. S. Wang Z. H. (2017b). Facile synthesis of flower-like Ag_3_VO_4_/Bi_2_WO_6_ heterojunction with enhanced visible-light photocatalytic activity. J. Colloid Interface Sci. 501, 156–163. 10.1016/j.jcis.2017.04.057 28453981

[B13] Li S. J. Hu S. W. Jiang W. Liu Y. P. Zhou Y. T. Liu Y.. (2018c). Hierarchical architectures of bismuth molybdate nanosheets onto nickel titanate nanofibers: facile synthesis and efficient photocatalytic removal of tetracycline hydrochloride. J. Colloid Interface Sci. 521, 42–49. 10.1016/j.jcis.2018.03.033 29549764

[B14] Li S. J. Hu S. W. Jiang W. Xu K. B. (2017c). One-pot solvothermal synthesis of Ag nanoparticles decorated BiOCOOH microflowers with enhanced visible light activity. Mater. Lett. 196, 343–346. 10.1016/j.matlet.2017.03.093

[B15] Li S. J. Hu S. W. Xu K. B. Jiang W. Liu Y. Leng Z.. (2017d). Construction of fiber-shaped silver oxide/tantalum nitride p-n heterojunctions as highly efficient visible-light-driven photocatalysts. J. Colloid Interface Sci. 504, 561–569. 10.1016/j.jcis.2017.06.018 28609739

[B16] Li S. J. Hu S. W. Zhang J. L. Jiang W. Liu J. S. (2017e). Facile synthesis of Fe_2_O_3_ nanoparticles anchored on Bi_2_MoO_6_ microflowers with improved visible light photocatalytic activity. J. Colloid Interface Sci. 497, 93–101. 10.1016/j.jcis.2017.02.069 28273515

[B17] Li S. J. Xu K. B. Hu S. W. Jiang W. Zhang J. L. Liu J. S. (2017f). Synthesis of flower-like Ag_2_O/BiOCOOH p-n heterojunction with enhanced visible light photocatalytic activity. Appl. Surf. Sci. 397, 95–103. 10.1016/j.apsusc.2016.11.081

[B18] Li X. Yu J. G. Jaroniec M. (2016). Hierarchical photocatalysts Chem. Soc.Rev. 45, 2603–2636. 10.1039/C5CS00838G 26963902

[B19] Li Z. Wu Z. Zhang S. Shen J. Feng W. Du Y. (2018e). Defect-sate of Indium doped bismuth molybdate nanosheets for enhanced photoreduction of chromium (VI) under visible light illumination. Dalton Trans. 47, 8110–8120. 10.1039/C8DT01807C 29878017

[B20] Liang J. Liu F. Deng J. Li M. Tong M. (2017). Efficient bacterial inactivation with Z-scheme AgI/Bi_2_MoO_6_ under visible light irradiation. Water Res. 123, 632–641. 10.1016/j.watres.2017.06.060 28709107

[B21] Liang J. Liu F. Li M. Liu W. Tong M. (2018). Facile synthesis of magnetic Fe_3_O_4_@BiOI@AgI for water decontamination with visible light irradiation: different mechanisms for different organic pollutants degradation and bacterial disinfection. Water Res. 137, 120–129. 10.1016/j.watres.2018.03.027 29547775

[B22] Lin W. Yu X. Zhu Y. Zhang Y. (2018). Graphene oxide/BiOCl nanocomposite films as efficient visible light photocatalysts. Front. Chem. 6:274. 10.3389/fchem.2018.00274 30137741 PMC6066524

[B23] Moroz P. Boddy A. Zamkov M. (2018). Challenges and prospects of photocatalytic applications based on semiconductor nanocrystals. Front. Chem. 6:353 10.3389/fchem.2018.00353 30159309 PMC6103974

[B24] Mousavi M. Habibi-Yangjeh A. Pouran S. R. (2018). Review on magnetically separable graphitic carbon nitride-based nanocomposites as promising visible-light-driven photocatalysts. J. Mater. Sci. Mater. Electron. 29, 1719–1747. 10.1007/s10854-017-8166-x

[B25] Ren M. Chen J. Wang P. Hou J. Qian J. Wang C.. (2018). Construction of silver iodide/silver/Bismuth Tantalate Z-scheme photocatalyst for effective visible light degradation of organic pollutants. J. Colloid Interface Sci. 532, 190–200. 10.1016/j.jcis.2018.07.141 30081264

[B26] Wang D. Astruc D. (2017). The recent development of efficient earth-abundant transition-metal nanocatalysts. Chem. Soc. Rev. 46, 816–854. 10.1039/C6CS00629A 28101543

[B27] Wang X. Yang J. Ma S. Zhao D. Dai J. Zhang D. (2016). In situ fabrication of AgIAgVO_3_ nanoribbon composites with enhanced visible photocatalytic activity for redox reactions. Catal. Sci. Technol. 6, 243–253. 10.1039/C5CY00787A

[B28] Wang Y. Wang H. Xu A. Song Z. (2018). Facile synthesis of Ag_3_PO_4_ modified with GQDs composites with enhanced visible-light photocatalytic activity. J. Mater. Sci. Mater. Electron. 29, 16691–16701. 10.1007/s10854-018-9762-0

[B29] Wang Z. H. Ma W. H. Chen C. C. Ji H. W. Zhao J. C. (2011). Probing paramagnetic species in titania-based heterogeneous photocatalysis by electron spin resonance (ESR) spectroscopy-A mini review. Chem. Eng. J. 170, 353–362. 10.1016/j.cej.2010.12.002

[B30] Wei C. Wang L. Dang L. Chen Q. Lu Q. Gao F. (2015). Bottom-up-then-up-down route for multi-level construction of hierarchical Bi_2_S_3_ superstructures with magnetism alteration. Sci. Rep. 5:10599. 10.1038/srep10599 26028331 PMC4450597

[B31] Wu M. J. Wu J. Z. Zhang J. Chen H. Zhou J. Z. Qian G. R. (2018). A review on fabricating heterostructures from layered double hydroxides for enhanced photocatalytic activities. Catal. Sci. Technol. 8, 1207–1228. 10.1039/C7CY02314F

[B32] Wu Z. Yu C. Liu R. Dionysiou D. D. Yang K. Wang C. (2017). Novel fluorinated Bi_2_MoO_6_ nanocrystals for efficient photocatalytic removal of water organic pollutants under different light source illumination. Appl. Catal. B-Environ. 209, 1–11. 10.1016/j.apcatb.2017.02.057

[B33] Xiang Z. Wang Y. Ju P. Long Y. Zhang D. (2018). Facile fabrication of AgI/BiVO_4_ composites with enhanced visible photocatalytic degradation and antibacterial ability. J. Alloys Compd. 721, 622–627. 10.1016/j.jallcom.2017.06.030

[B34] Xiong J. Y. Cheng G. Lu Z. Tang J. L. Yu X. L. Chen R. (2011). BiOCOOH hierarchical nanostructures: shape-controlled solvothermal synthesis and photocatalytic degradation performances. Cryst. Eng. Comm. 13, 2381–2390. 10.1039/c0ce00705f

[B35] Xu B. Y. An Y. Liu Y. Y. Huang B. B. Qin X. Y. Zhang X. Y.. (2016). An efficient visible-light photocatalyst made from a nonpolar layered semiconductor by grafting electron-withdrawing organic molecules to its surface. Chem. Commun. 52, 13507–13510. 10.1039/C6CC07849D 27803947

[B36] Xu J. Wang Y. Chen M. Teng F. (2018). A novel BiOCl/BiOCOOH heterojunction photocatalyst with significantly enhanced photocatalytic activity. Mater. Lett. 222, 176–179. 10.1016/j.matlet.2018.04.002

[B37] Ye R. Zhao J. Wickemeyer B. B. Toste F. D. Somorjai G. A. (2018). Foundations and strategies of the construction of hybrid catalysts for optimized performances. Nat. Catal. 1, 318–325. 10.1038/s41929-018-0052-2

[B38] Yuan X. Wu Z. Zeng G. Jiang L. Zhang J. Xiong T. (2018). Synthesis and boosting visible light photoactivity of Ag@AgI/CdWO_4_ towards refractory organic pollutants degradation based on interfacialcharge transfer. App. Surf. Sci. 454, 293–304. 10.1016/j.apsusc.2018.05.163

[B39] Zhang G. G. Lan Z. A. Wang X. C. (2016). Conjugated polymers: catalysts for photocatalytic hydrogen evolution. Angew. Chem. Int. Ed. 55, 15712–15727. 10.1002/anie.201607375 27528426

[B40] Zhang J. L. Ma Z. (2018). Ag-Ag_2_CO_3_/Bi_2_MoO_6_ composites with enhanced visible-light-driven catalytic activity. J. Taiwan Inst. Chem. Eng. 71, 156–164. 10.1016/j.jtice.2016.11.030

[B41] Zhang L. Zhang Q. Xie H. Guo J. Lyu H. Li Y. (2017). Electrospun titania nanofibers segregated by graphene oxide for improved visible light photocatalysis. Appl. Catal. B Environ. 201, 470–478. 10.1016/j.apcatb.2016.08.056

[B42] Zhang M. Y. Shao C. L. Mu J. B. Zhang Z. Y. Guo Z. C. Zhang P. (2012). One-dimensional Bi_2_MoO_6_/TiO_2_ hierarchical heterostructures with enhanced photocatalytic activity. CrystEngComm 14, 605–612. 10.1039/C1CE05974B

[B43] Zhang W. Sun Y. Xiao Z. Li W. Li B. Huang X. (2015). Heterostructures of CuS nanoparticle/ZnO nanorod arrays on carbon fibers with improved visible and solar light photocatalytic properties. J. Mater. Chem. A 3, 7304–7313. 10.1039/C5TA00560D

[B44] Zhu S. S. Wang D. W. (2017). Photocatalysis: basic principles, diverse forms of implementations and emerging scientifc opportunities. Adv. Energy Mater. 7:1700841 10.1002/aenm.201700841

