# Supplementary material for: Synthesis of Flower-Like AgI/BiOCOOH p-n Heterojunctions With Enhanced Visible-Light Photocatalytic Performance for the Removal of Toxic Pollutants
Source: Front Chem. 2018 Oct 26;6:518. doi: 10.3389/fchem.2018.00518 (PMC6213583; doi:10.3389/fchem.2018.00518)
Supplement: Supplementary file 1 [file Data_Sheet_1.doc]

**Supplementary Data**

**Synthesis of flower-like AgI/BiOCOOH p-n heterojunctions with enhanced visible-light photocatalytic performance for the removal of toxic pollutants**

Shijie Li^1*^, Wei Jiang^1^, Kaibing Xu^2^*, Shiwei Hu^1^, Yu Liu^1^, Yingtang Zhou^1^, Jianshe Liu^3^

^1^ Key Laboratory of key technical factors in Zhejiang seafood health hazards, Institute of Innovation & Application, Zhejiang Ocean University, Zhoushan, Zhejiang Province, 316022, China.

^2^ State Key Laboratory for Modification of Chemical Fibers and Polymer Materials, Research Center for Analysis and Measurement, Donghua University, Shanghai, 201620, China

^3^ State Environmental Protection Engineering Center for Pollution Treatment and Control in Textile Industry, College of Environmental Science and Engineering, Donghua University, Shanghai 201620, China.

* Email address: [lishijie@zjou.edu.cn](mailto:lishijie@zjou.edu.cn) (S. Li); xukaibing@dhu.edu.cn (K. Xu)

**Table 1** BET surface areas of samples

| Samples | BiOCOOH | S1 | S2 | S3 | S4 |  |
| --- | --- | --- | --- | --- | --- | --- |
| BET(m^2^g^-1^) | 26.13 | 28.27 | 24.26 | 20.72 | 15.21 |  |

**Figures**

**
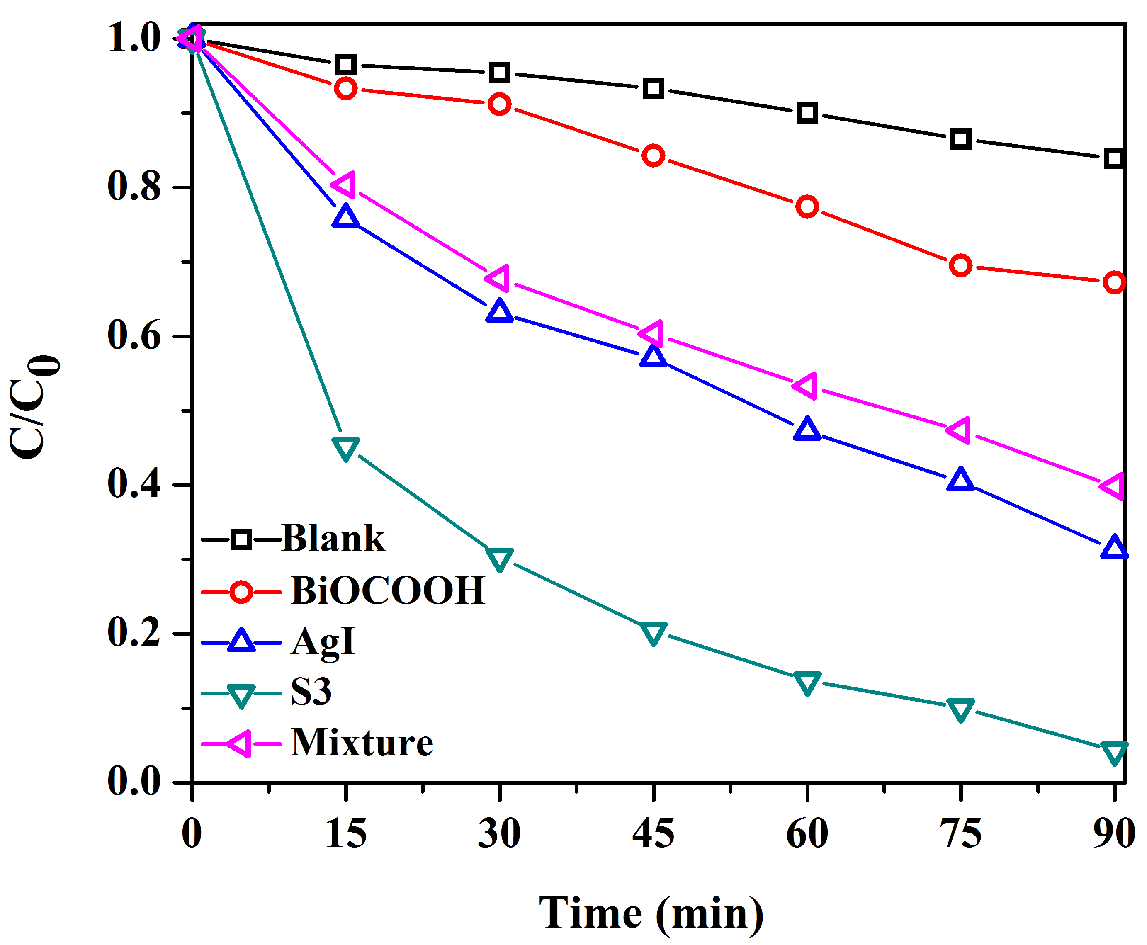
**

**Figure S1.** The MB degradation curves over different samples.

**
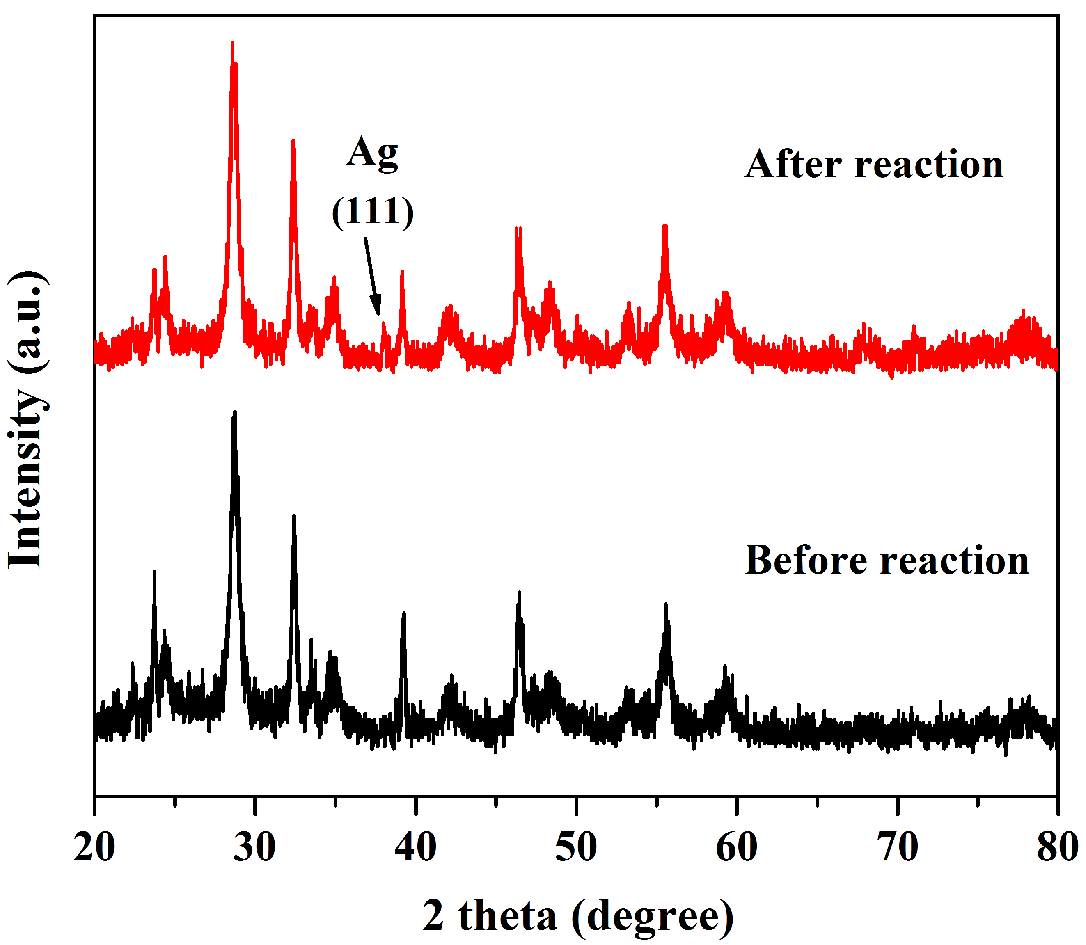
**

**Figure S2.** The XRD patterns of S3 before and after 6 successive runs.
